# Early-Stage Loss of GALNT6 Predicts Poor Clinical Outcome in Colorectal Cancer

**DOI:** 10.3389/fonc.2022.802548

**Published:** 2022-05-27

**Authors:** Makiko Ogawa, Atsushi Tanaka, Kei Namba, Jinru Shia, Julia Y. Wang, Michael H. Roehrl

**Affiliations:** ^1^Department of Pathology and Laboratory Medicine, Memorial Sloan Kettering Cancer Center, New York, NY, United States; ^2^Human Oncology and Pathogenesis Program, Memorial Sloan Kettering Cancer Center, New York, NY, United States; ^3^Department of Thoracic Surgery and Breast and Endocrine Surgery, Okayama University Graduate School of Medicine, Dentistry and Pharmaceutical Sciences, Okayama, Japan; ^4^Curandis, New York, NY, United States

**Keywords:** GALNT6, glycosylation, colorectal cancer, early stage, risk prediction

## Abstract

Colorectal adenocarcinomas arise from luminal lining epithelium of the colorectal tract which is covered with highly glycosylated mucins. Mucin O-glycosylation is initiated by a family of polypeptide N-acteylgalactosaminyltransferases (GALNTs). This study examined GALNT6 protein expression in 679 colorectal tumors, including 574 early-stage and 105 late-stage cancers. GALNT6 expression in cancer tissue varied widely between patients ranging from high levels to complete loss. Loss of GALNT6 occurred in 9.9% of early-stage and 15.2% of late-stage cancers and was more prevalent in grade 3 or MSI subtype tumors. Survival analyses revealed that loss of GALNT6 expression is prognostic of reduced overall survival, and univariate and multivariate analyses demonstrated that loss of GALNT6 is an independent risk variable. We also analyzed 508-case TCGA and 63-case CPTAC colorectal cancer cohorts for all members of the GALNT enzyme family, the mucin family, as well as KRAS and BRAF mutations. GLANT6 mRNA expression showed no strong correlation with other GALNTs or mucins but was significantly higher in KRAS mutated or BRAF wild-type early-stage cancers. Using large cohorts of patients and different approaches, this study shows that loss of GALNT6 enzyme in early-stage colorectal cancer predicts poor clinical outcomes.

## Introduction

Colorectal cancer is among the most common cancers diagnosed in both men and women. According to the American Cancer Society, colorectal cancer is the third leading cause of cancer-related deaths in the United States, and the lifetime risk of developing colorectal cancer is about 1 in 23 (4.3%) for men and 1 in 25 (4.0%) for women. Colorectal cancer is curable if detected early, and, in fact, the death rate from colorectal cancer has been dropping in recent years, partially due to increased screening efforts and early detection. However, early-onset disease has been on the rise in patients younger than age 50, and these patients are often diagnosed at an advanced stage, which poses numerous unique challenges for cancer management (1). Hence, there is an urgent need for molecular biomarkers that are readily detectable and can be used for better risk stratification of early-stage tumors.

Colorectal cancer originates from the inner lining of the colorectal tract epithelium, which is covered by a mucus layer composed of highly glycosylated proteins called mucins. This mucus layer provides lubrication for the passage of food and waste, protects the host epithelium from commensal microorganisms and invading pathogens, and participates in cell signaling pathways (2). Altered mucin-glycosylation has significant impact on host immunomodulation, anti-tumor immunity, and gut-microbiota interaction (3). Cancer-associated mucins show antigenic differences from normal mucins, and aberrant mucin O-glycosylation in cancer gives rise to tumor-associated antigens such as the Tn determinant (alpha-GalNAc-O-Ser/Thr) that has been explored as a potential target for immunotherapy (4, 5). In colorectal cancer, 65 of 78 (83%) of patients expressed the Tn antigen (6). Aberrant O-glycosylation of MUC1 and MUC4 has been detected in colorectal cancer, which results in unique antigenic epitopes that induce cancer-associated autoantibodies (7).

Mucin-type O-glycosylation, an evolutionarily conserved and essential post-translational protein modification, is controlled by a large family of UDP-N-acetyl-alpha-D-galactoasmine:polypeptide N-acetylgalactosaminyltransferase (GALNT or GalNAc-T) enzymes (8–10). They initiate O-linked glycosylation in the Golgi apparatus by catalyzing the transfer of an N-acetyl-D-galactosamine (GalNAc) onto a serine or threonine residue in the target protein (11). GalNAc-Ts are the largest glycosyltransferase enzyme family catalyzing a single known glycosidic linkage, and they have different but overlapping substrate specificities and patterns of expression (12–15). In particular, N-acetylgalatosaminyltransferase 6 (GALNT6) has been found to be differentially expressed in various cancers (16–27). In colorectal cancer, GALNT6 is identified as one of the susceptibility genes (28), and GALNT6 expression is involved in oncogenic transformation and progression (29, 30). Given its crucial function in O-glycosylation of the colorectal mucus layer, we investigated GALNT6 as a potential marker of colorectal cancer, particularly of early-stage disease.

## Materials and Methods

### Clinical Case Selection and Pathological Data

Colorectal cancer tissue specimens from 679 patients were obtained from the Precision Pathology Biobank of Memorial Sloan Kettering Cancer Center (MSKCC). The cohort comprises 574 cases of early stage (AJCC stages I or II) and 105 cases of late stage (AJCC stages III or IV). These tumor tissues had been surgically resected at MSKCC between 1981 to 2000. The study was approved by MSKCC’s Institutional Review Board, and clinical data were acquired retrospectively in an anonymized manner. Clinical parameters, including patient age, treatment history, recurrence, and survival status, were retrieved from medical records. Histologic type and other clinicopathological parameters of all samples were re-verified by gastrointestinal subspecialty pathologists.

### Tissue Microarray Construction

Tissue microarrays were constructed from the 679 colorectal tumors. All archival tissue specimens had been fixed with formalin and embedded in paraffin blocks. Three 2-mm tissue cores were drilled out from each donor paraffin tissue block and transferred to tissue array blocks using a TMA Grand Master robot (3DHistech). The cored areas were defined by a certified pathologist for each case and tissue block and included tumor tissue as well as normal mucosal tissue.

### Immunohistochemistry (IHC)

The tissue microarray blocks were cut into 4-µm sections. Paraffin was removed with xylene, and antigens were retrieved using BOND epitope retrieval solution 1 (citrate buffer, pH 6.0; Leica) on a Leica BOND RX slide stainer for 30 min at 100 °C. Tissue sections were incubated with GALNT6-specific polyclonal antibodies (HPA011762, 1:150, Atlas Antibodies, Sigma) for 30 min, followed by visualization with the Leica Bond detection kit.

### Immunohistochemical Scoring

Stained IHC tissue slides were evaluated independently by two pathologists without knowledge of the patients’ clinical information. Each tissue section was scored by counting the number of cancer cells staining positively for GALNT6 protein (staining intensity ≥1+) relative to the total number of evaluated cancer cells. A minimum of 500 cancer cells were evaluated per tissue sample. A tissue sample was considered positive for GALNT6 staining when ≥10% of tumor cells showed granular cytoplasmic staining (26, 31).

### TCGA and CPTAC Dataset Analysis

A 508-case colorectal cancer cohort from the Cancer Genome Atlas (TCGA) (32) and a 63-case colorectal cancer cohort from the Clinical Proteomics Tumor Assessment Consortium (CPTAC) (33) were analyzed for gene expression correlations between GALNT6 expression and mucin (MUC) genes, all members of the GALNT enzyme family, KRAS mutation status, or BRAF mutation status. The sequencing results and relevant clinical information of the cohorts were downloaded from cBioPortal (*https://www.cbioportal.org/*).

### Statistical Analysis

Categorical variables were compared using Fisher’s exact test. Survival analyses were conducted using the Kaplan-Meier method and compared by a log-rank test. Multivariate analyses of prognostic factors were performed with logistic regression models by using factors that showed significant differences (*p*<0.05) in univariate analyses. A backward elimination method was used to select variables for the final model. Correlation coefficients were calculated by the Spearman method. Statistical analyses were performed using JMP Pro 14 software (SAS).

## Results

### GALNT6 Expression Pattern in Colorectal Cancer

**Figure 1:**
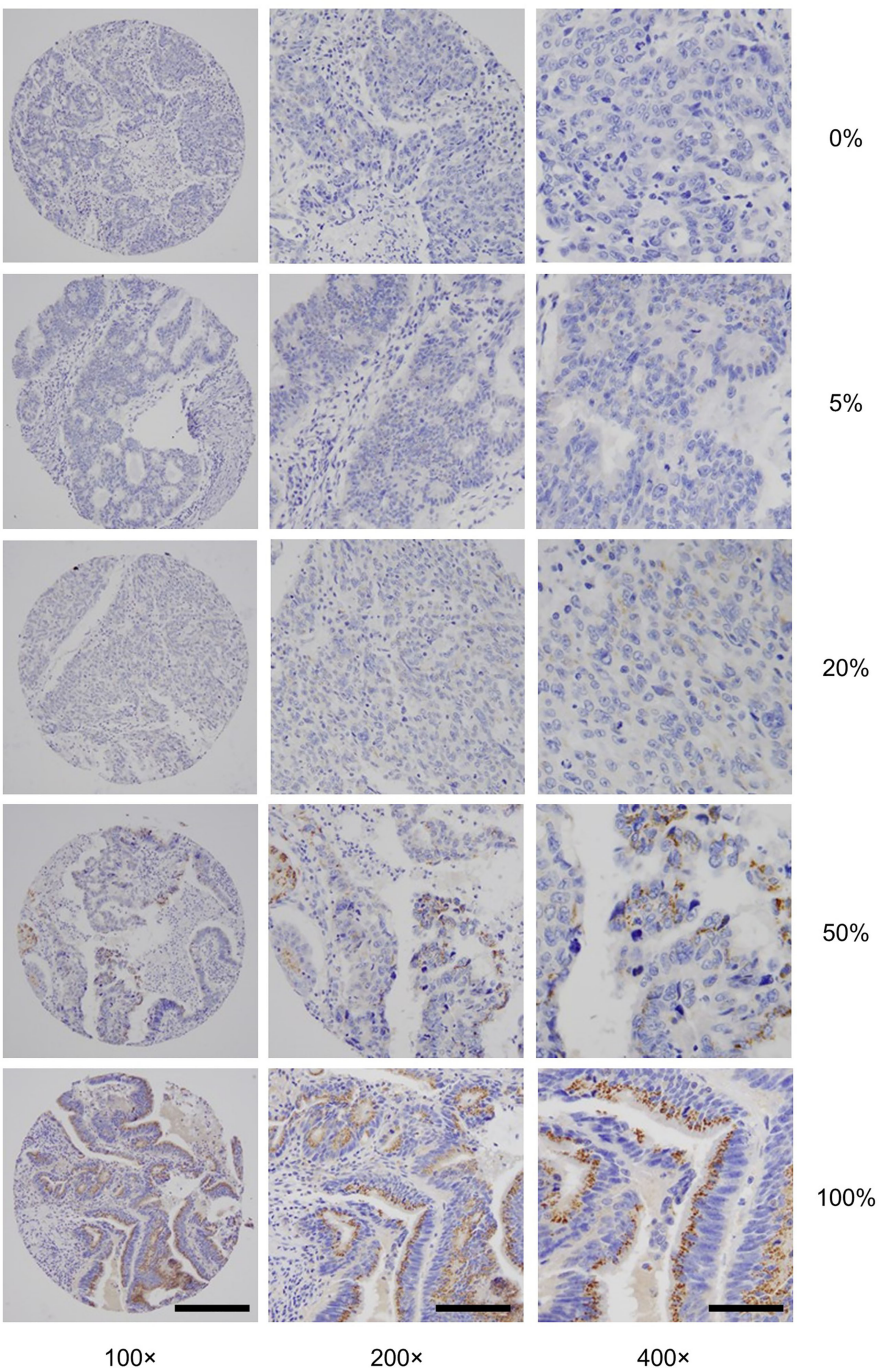
Representative GALNT6 protein expression in five different colorectal adenocarcinomas (rows from top to bottom). GALNT6 protein expression (brown) with nuclei counter-stained (blue). The representative cases shown correspond to histologically moderately (rows 1, 2, 4, and 5) or poorly (row 4) differentiated adenocarcinomas. Numbers to the right indicate the percentages of GALNT6 positive tumor cells for each case. The final microscopic magnification is shown at the bottom of each column. The scale bars represent, from left to right columns, 120, 60, and 30 µm, respectively.

**Figure 2:**
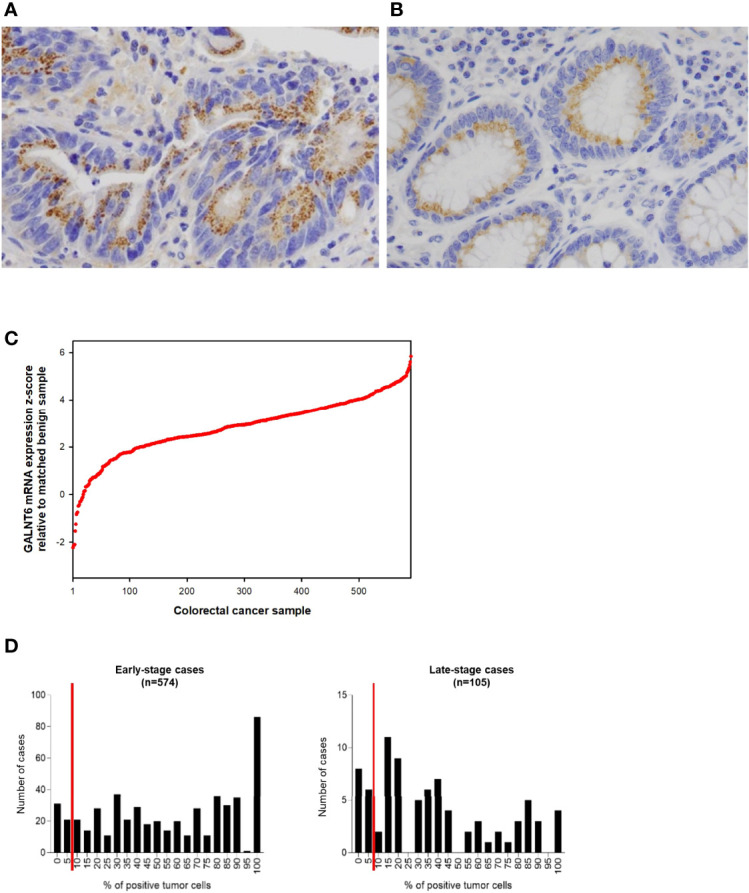
Examples of **(A)** a case of colorectal adenocarcinoma with high % of tumor cells expressing GALNT6 protein and **(B)** of GALNT6 protein expression in normal/benign colonic mucosa. GALNT6 proteins are colored brown with nuclei counterstained in blue. **(C)** GALNT6 mRNA z-scores of colorectal adenocarcinomas (relative to matched benign mucosa) from the TCGA cohort sorted in ascending order (a positive z-score corresponds to higher transcript expression in the cancer relative to matched normal). **(D)** Distribution of GALNT6 protein expression (% of tumor cells staining positive) in colorectal cancer. Tissues with ≥10% of tumor cells with GALNT6 immunohistochemical staining are considered positive (red lines show cut-off). There are fewer GALNT6-negative cases among early stage (stages I and II, 9.9% of cases) vs. late stage (stages III and IV, 15.2% of cases) cancers.

To gain insights into GALNT6 protein expression in colorectal cancers, we examined 679 tumor specimens in tissue microarrays by immunohistochemistry. Representative immunohistochemical staining patterns of GALNT6 are shown in **Figure 1**. GALNT6 expression intensity varies greatly between these specimens, ranging from no cells to virtually all tumor cells expressing the enzyme. When expressed by cancer cells, GALNT6 expression is mostly cytoplasmic in a granular pattern near the nucleus. When cancer cells retain secretory polarity, GALNT6 is expressed on the luminal/apical side (**Figure 2A**), similar to expression observed in benign enterocytes (**Figure 2B**).

GALNT6 protein is expressed by benign crypt enterocytes, particularly in the apical cytoplasmic portion of enterocytes, in a location between the nucleus and luminal mucin, consistent with a Golgi-associated location of GALNT6. There appears to be some variability of GALNT6 protein expression between different crypts, which could be due to either expression variability or the limited amount of visible cytoplasm in cells with large luminal mucin compartments. We used the TCGA cohort to assess the relative abundance differences of GALNT6 mRNA between colorectal adenocarcinomas and matched normal mucosa (**Figure 2C**). GALNT6 mRNA is increased in a majority of carcinomas (median z-score, +2.922; 25^th^/75^th^ percentile z-scores, +2.182/+3.684).

Since the number of GALNT6-expressing tumor cells varied widely between different patients’ cancers (and much more than staining intensity/abundance per cell), we took a semi-quantitative approach and determined the fraction of GALNT6-positive tumor cells for all cases in our cohort (**Figure 2D**). Based on immunohistochemical staining distribution, we defined GALNT6 positivity as ≥10% of tumor cells showing GALNT6 expression and, conversely, loss of GALNT6 expression as cases with <10% of tumor cells staining. Among all cases, 10.8% (73/679) of patients displayed loss of GALNT6 protein. Judging by the expression distributions, GALNT6 expression appears to decrease as the cancers advance, and cases of late-stage cancer are more likely to show negative or reduced GALNT6 expression (**Figure 2D**). While 9.9% (57/574) of early-stage colorectal cancer cases were negative for GALNT6 expression, 15.2% (16/105) of late-stage cancers were GALNT6-negative.

### GALNT6 Expression vs. Clinicopathological Features

To further understand GALNT6 expression in colorectal cancer, we investigated its relation with a number of clinicopathological features, including patient age and gender, histologic type, tumor grade, location, lymphovascular and perineural invasion, pTNM stage, and MSI/MSS subtype (**Table 1**). The 679-case cohort of this study was carefully selected to reflect general disease distribution. The cohort is evenly distributed in terms of patient gender, including 51.5% (350/679) males and 48.5% (329/679) females. There are 616 cases (90.7%) of low-grade (G1 and G2) tumors and 63 cases (9.3%) of poorly differentiated G3 tumors. Furthermore, 78.5% (533/679) of these cases had intact mismatch repair status and were categorized as microsatellite stable (MSS), while 21.5% (146/679) had microsatellite instability (MSI).

**Table 1:** GALNT6 protein expression vs. clinical features of colorectal cancer cases.

	Early-stage CRC (n = 574)		p-value (two-tailed)	Late-stage CRC (n = 105)		p-value (two-tailed)
	Positive*	Negative*		Positive*	Negative*	
Total	517 (90.1%)	57 (9.9%)		89 (84.8%)	16 (15.2%)	
Gender			0.6765			0.2820
Male	271 (90.6%)	28 (9.4%)		41 (80.4%)	10 (19.6%)	
Female	246 (89.5%)	29 (10.5%)		48 (88.9%)	6 (11.1%)	
Age (years)			1.0000			0.2888
≤70	301 (90.1%)	33 (9.9%)		84 (85.7%)	14 (14.3%)	
>70	216 (90.0%)	24 (10.0%)		5 (71.4%)	2 (28.6%)	
Histology			0.2022			0.6734
Not mucinous	478 (90.5%)	50 (9.5%)		80 (85.1%)	14 (14.9%)	
Mucinous	39 (84.8%)	7 (15.2%)		9 (81.8%)	2 (18.2%)	
Tumor differentiation			<0.0001			0.0009
G1/G2	486 (92.6%)	39 (7.4%)		82 (90.1%)	9 (9.9%)	
G3	31 (63.3%)	18 (36.7%)		7 (50.0%)	7 (50.0%)	
Location			0.0361			0.1638
Left	259 (92.8%)	20 (7.2%)		61 (88.4%)	8 (11.6%)	
Right	258 (87.5%)	37 (12.5%)		28 (77.8%)	8 (22.2%)	
Lymphovascular invasion			0.0117			0.1454
Absent	456 (91.4%)	43 (8.6%)		28 (93.3%)	2 (6.7%)	
Present	61 (81.3%)	14 (18.7%)		61 (81.3%)	14 (18.7%)	
Perineural invasion			0.1944			0.1605
Absent	493 (90.5%)	52 (9.5%)		58 (89.2%)	7 (10.8%)	
Present	24 (82.8%)	5 (17.2%)		31 (77.5%)	9 (22.5%)	
Disease stage			0.4699			0.0359
I	194 (91.5%)	18 (8.5%)				
II	323 (89.2%)	39 (10.8%)				
III				60 (80.0%)	15 (20.0%)	
IV				29 (96.7%)	1 (3.3%)	
MMR status			<0.0001			0.0045
Intact (MSS)	419 (95.0%)	22 (5.0%)		82 (89.1%)	10 (10.9%)	
Lost (MSI)	98 (73.7%)	35 (26.3%)		7 (53.8%)	6 (46.2%)	

*Positive cut-off set at ≥10% positive cells.

In both early stage and late-stage groups, GALN6 expression status did not differ significantly in terms of patient gender or age, tumor histology (mucinous vs. not mucinous), or perineural invasion status (**Table 1**). However, loss of GALNT6 expression was significantly correlated with high tumor grade. Among G3 tumors, 36.7% of early stage and 50.0% of late-stage cases were GALNT6-negative, whereas only 7.4% of early-stage and 9.9% of late-stage G1/G2 tumors were GALNT6-negative. In early-stage cases, loss of GALNT6 expression is more pronounced in right-sided tumors and in tumors with lymphovascular invasion. Intriguingly, GALNT6 loss did not differ significantly among early-stage cancers (stage I, 8.5%; stage II, 10.8%), whereas, among late-stage cancers, stage III cancers had a significantly higher rate of GALNT6 loss (20.0%) than stage IV cancers (3.3%). Furthermore, GALNT6-negative cases are more prevalent in the MSI subtype than in the MSS subtype of colorectal cancer. Among early-stage cancer cases, 26.3% (35/133) of MSI tumors were GALNT6-negative but only 5.0% (22/441) of MSS tumors were GLANT6-negative. Among late-stage cancer cases, 46.2% (6/13) of MSI tumors and 10.9% (10/92) MSS tumors were GALNT6-negative.

### Loss of GALNT6 Protein Expression Correlates With Short Survival Time in Early-Stage Colorectal Cancer

**Figure 3:**
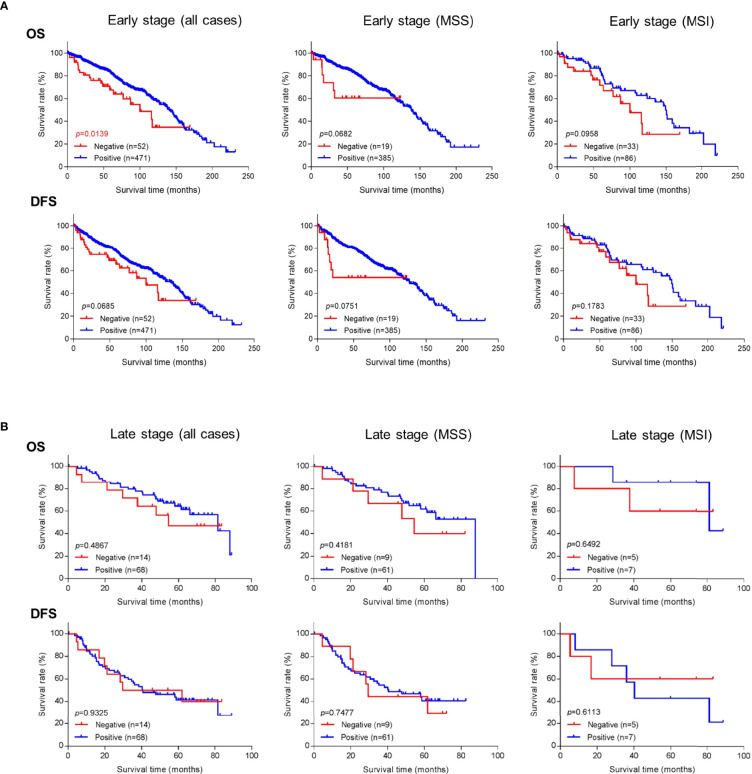
Overall survival (OS) or disease-free survival (DFS) in **(A)** early-stage and **(B)** late-stage colorectal cancer as a function of GALNT6 protein expression status (positive/retained or negative/lost). Loss of GALNT6 is associated with shorter survival in early-stage disease.

Because better risk stratification is particularly crucial for treatment decisions in early-stage cancer, we examined whether GALNT6 protein expression is potentially prognostic. Among all 574 patients with early-stage colorectal cancer, 523 cases had available follow-up survival data, had not received neoadjuvant therapy prior to surgery, and were thus further analyzed. Mean and median clinical follow-up periods for this cohort were 80.2 and 71.9 months, respectively. Kaplan-Meier analyses revealed that early-stage patients with GALNT6 protein loss had significantly shorter overall survival than those with positive GALNT6 expression (*p*=0.0139) (**Figure 3A**). GALNT6-negative patients also had shorter disease-free survival, although the difference is not statistically significant. When early-stage MSI and MSS subtypes were examined separately, GALNT6-negative patients had a trend for shorter overall survival and disease-free survival times than those with retained expression, although the differences were not statistically significant, perhaps due to smaller case numbers in each subtype (**Figure 3A**).

Negative vs. positive GALTN6 protein expression did not show statistical correlations with overall survival and disease-free survival among patients with late-stage colorectal cancers, although a trend for lower overall survival was observed for GALNT6 loss (**Figure 3B**). No dependence on MSI or MSS subtype status was detectable.

To further investigate whether GALNT6 is an independent prognostic factor in early-stage colorectal cancer, we performed both univariate and multivariate analyses (**Table 2**). Both analyses revealed that loss of GALNT6 protein expression was an independent indicator for poor overall survival. In addition, patient age, lymphovascular invasion, and AJCC stage are factors differentiating patient survival. Other clinical features, such as gender, tumor location, histology, tumor grade, perineural invasion, and MMR status, did not significantly correlate with clinical survival in a multivariate model (**Table 2**). Hence, these statistical analyses further support GALNT6 protein as a prognostic marker for poor survival in early-stage colorectal cancer.

**Table 2:** Prognostic potential of GALNT6 protein expression in early-stage colorectal cancer.

Variable	Overall survival				Disease-free survival	
	Univariate*		Multivariate*		Univariate*	
	HR (95% CI)	p-value	HR (95% CI)	p-value	HR (95% CI)	p-value
Gender	1.17	0.2584			1.30	0.0532
(male vs. female)	(0.89-1.55)				(1.00-1.70)	
Age (years)	2.64	<0.0001	2.61	<0.0001	2.23	<0.0001
(>70 vs. ≤70)	(1.99-3.60)		(1.95-3.50)		(1.71-2.93)	
Tumor location	1.23	0.1394			1.12	0.3969
(right vs. left)	(0.93-1.63)				(0.86-1.46)	
Histology	0.68	0.1484			0.73	0.2107
(mucinous vs. other)	(0.38-1.13)				(0.42-1.18)	
Tumor differentiation	1.25	0.4188			1.12	0.6601
(G3 vs. G1/G2)	(0.71-2.05)				(0.65-1.81)	
Lymphovascular invasion	1.77	0.0073	1.77	0.0083	2.01	0.0004
	(1.18-2.57)		(1.17-2.60)		(1.39-2.83)	
Perineural invasion	2.12	0.0153			2.04	0.0164
	(1.17-3.55)				(1.15-3.35)	
Disease stage	1.84	<0.0001	1.57	0.0028	1.88	<0.0001
(II vs. I)	(1.36-2.50)		(1.17-2.15)		(1.42-2.53)	
MMR	1.02	0.9017			0.92	0.5896
(lost vs. intact)	(0.73-1.40)				(0.66-1.25)	
GALNT6 expression	0.56	0.0242	0.60	0.0482	0.66	0.0867
(positive vs. negative)	(0.36-0.92)		(0.38-1.00)		(0.43-1.07)	

*HR, hazard ratio; CI, confidence interval; two-tailed p-value.

## Discussion

Our study investigated a very large cohort of well-characterized early-stage colorectal cancer patients (574 cases) who had not undergone pre-surgical neoadjuvant therapy. We found that loss of GALNT6 protein expression, as defined by <10% of tumor cells expressing GALNT6, predicts poor overall survival in early-stage colorectal cancer patients (stages I and II). Our findings are generally in line with previous studies. In a cohort of 84 colorectal cancer tissues and 77 normal non-tumor mucosal tissues, cancer patients with higher expression of GALNT6 protein had better overall survivals than those with lower expression (30). In another study of 195 patients with stage II and III colorectal cancer, tumors lacking GALNT6 protein were associated with poorer histologic differentiation, and patients with negative GALNT6 had significantly poorer disease-free survival and overall survival (31). Lack of GALNT6 protein expression was also associated with poor therapeutic response to 5-FU-based adjuvant chemotherapy in colorectal cancer (31). In a cohort of 81 colon cancer specimens, patients expressing GALNT6 had a significantly increased overall survival compared with GALNT6-negative patients (26).

Because loss of protein expression of GALNT6 is prognostic in early-stage cancer patients, we asked whether a similar trend would be observed at the level of GALNT6 mRNA expression. We examined a 508-case TCGA cohort of colorectal cancers. Interestingly, however, GALNT6 mRNA expression levels did not show significant correlation with patient survival times. It is known that mRNA expression levels are frequently not concordant with protein expression levels, which may explain why GALNT6 protein expression is prognostic whereas its mRNA expression is not.

**Figure 4:**
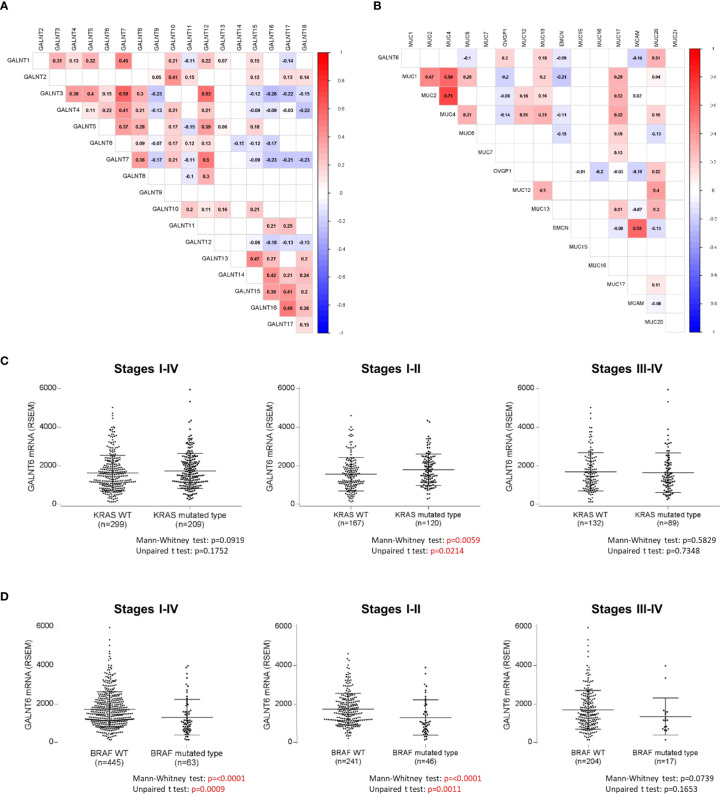
Pairwise comparison of mRNA expression levels of **(A)** GALNT6 with GALNT gene family members and **(B)** GALNT6 with MUC mucin gene family members (positive and negative correlation coefficients are shown). Comparison of GALNT6 mRNA expression levels as a function of **(C)** KRAS mutations status or **(D)** BRAF mutation status. All data based on a 508-case TCGA colorectal cancer cohort.

The functional role of GALNT6 in colorectal cancer remains unknown at present. Since GALNT6 belongs to a large family of N-acetylgalactosaminytransferases that initiate mucin-type O-glycosylation, we asked whether GALNT family enzymes are redundant in colorectal cancer. We examined the mRNA expression of all GALNTs in the 508-case TCGA cohort of colorectal cancer (**Figure 4A**). GALTN3, GALNT7, and GALN12 showed strong correlation between each other. The abundance of GALNT1 and GALNT2 has been found to be significantly higher in colorectal cancer than that in normal epithelium (34), and high GALNT3 protein expression has been reported to be an indicator of tumor differentiation and a prognostic factor in colorectal cancer (35). However, GALNT6 did not show any correlation with either GALNT1 or GALNT2 and only weak positive correlation with GALNT3 (**Figure 4A**). In addition, GALNT6 did not show strong positive gene expression correlation with any of the other GALNTs. These findings suggest that GALNT6 may be functionally unique and not redundant with other GALNTs in colorectal cancer.

The endogenous substrates of GALTN6 in colorectal cancer are currently unknown. Glycosylation is strongly substrate-, site-, and context-specific, leading to the formation of highly complex glycan structures. Altered expression of GalNAc-T enzymes can perturb the cellular glycan structures and severely hinder the normal function of the substrate proteins. Since mucins are abundant in the lower intestinal tract and constitute major substrates of GALNT enzymes, we studied correlations between GALNT6 and mucins (MUC) genes using the 508-case TCGA cancer dataset. GALNT6 mRNA expression showed weakly positive correlation with MUC20, OVGP1 (MUC9), and MUC13 and weakly negative correlation with MCAM (MUC18) and MUC6, but no correlations with other mucins (**Figure 4B**). MUC13 has been found to be highly expressed by human colorectal carcinomas as demonstrated by immunochemistry in 99 colorectal cancer cases (36). It will be interesting to study whether colorectal cancers with loss of GALNT6 might show elevated expression of the negatively correlated mucins.

Since KRAS and BRAF gene mutations are frequently present in colon cancers, we asked whether KRAS and BRAF mutations were more prevalent in GALNT6-negative tumors. We examined the 508-case TCGA cohort and a second 63-case CPTAC colorectal cancer cohort (33). Interestingly, GALNT6 mRNA expression showed significant correlation with KRAS and BRAF status in early-stage colorectal cancers (**Figures 4C, D**). KRAS wild-type cancers had lower GALNT6 mRNA expression levels than KRAS mutated cancers, whereas BRAF mutated tumors had lower GALNT6 mRNA expression levels than BRAF wild-type tumors. This difference, however, was not seen at protein level. Analysis of the peptide spectrum counts in the 63-case CPTAC cohort did not reveal significant correlations between GALNT6 protein expression levels and BRAF or KRAS mutation status (data not shown). Any possible functional cross-talk between GALNT6 and KRAS/BRAF will require further study.

In summary, our study shows that loss of the GALNT6 enzyme occurs in a subset of early-stage colorectal cancer patients and is significantly associated with poor (G3) differentiation, right-sided location, lymphovascular invasion, MSI subtype, and shorter overall survival. Based on various statistical analyses of a large patient cohort, loss of GALNT6 protein expression could potentially serve as a prognostic marker to risk-stratify cancers of early clinical stage (stages I and II) and aid in selecting patients for more intensive adjuvant therapy or closer surveillance.

## Data Availability Statement

The raw data supporting the conclusions of this article will be made available by the authors, without undue reservation.

## Ethics Statement

The studies involving human participants were reviewed and approved by MSKCC's Institutional Review Board (IRB). Written informed consent for participation was not required for this study in accordance with national legislation and institutional requirements.

## Author Contributions

MO and AT carried out experiments and analyses. KN assisted with analyses. JS provided reagents and advice. JW assisted with data analysis and manuscript preparation. MR directed the study. All authors contributed to the article and approved the submitted version.

## Funding

This study was supported in part by funding from the Farmer Family Foundation. MR acknowledges a Cycle for Survival Equinox innovation grant, an investigator grant from the Neuroendocrine Tumor Research Foundation (NETRF), NCI CPTAC contract 17X173, and NIH/NCI grants and NIH/NCI grants R21 CA251992 and R21 CA263262. This research was funded in part through the MSKCC NIH/NCI Cancer Center Support Grant P30 CA008748. The funders had no role in study design, data collection and analysis, decision to publish, or preparation of the manuscript.

## Conflict of Interest

JW is founder and equity holder of Curandis. MR is member of the Scientific Advisory Boards of Azenta and Universal DX. None of these companies had any influence in support, design, execution, data analysis, or any other aspect of this study.

The remaining authors declare that the research was conducted in the absence of any commercial or financial relationships that could be construed as a potential conflict of interest.

## Publisher’s Note

All claims expressed in this article are solely those of the authors and do not necessarily represent those of their affiliated organizations, or those of the publisher, the editors and the reviewers. Any product that may be evaluated in this article, or claim that may be made by its manufacturer, is not guaranteed or endorsed by the publisher.
